# Cross-sectional and prospective associations between behavioural patterns and adiposity in school-aged children

**DOI:** 10.1017/S136898002300112X

**Published:** 2023-09

**Authors:** Ninoshka J D’Souza, Katherine Downing, Miaobing Zheng, Gavin Abbott, Sandrine Lioret, Karen J Campbell, Kylie D Hesketh

**Affiliations:** 1 Institute for Physical Activity and Nutrition, School of Exercise and Nutrition Sciences, Deakin University, Burwood 3125, VIC, Australia; 2 Research Center in Epidemiology and Biostatistics (CRESS), Université de Paris, INSERM, INRAE, 75004 Paris, France

**Keywords:** Diet, Physical activity, Sedentary behaviour, Sleep, Children, Adiposity

## Abstract

**Objective:**

Behavioural patterns are important in understanding the synergistic effect of multiple health behaviours on childhood adiposity. Most previous evidence assessing associations between patterns and adiposity were cross-sectional and investigated two or three behaviour domains within patterns. This study aimed to identify behavioural patterns comprising four behaviour domains and investigate associations with adiposity risk in children.

**Design:**

Parent-report and accelerometry data were used to capture daily dietary, physical activity, sedentary behaviour and sleep data. Variables were standardised and included in the latent profile analysis to derive behavioural patterns. Trained researchers measured children’s height, weight and waist circumference using standardised protocols. Associations of patterns and adiposity measures were tested using multiple linear regression.

**Setting:**

Melbourne, Australia.

**Participants:**

A total of 337 children followed up at 6–8 years (T2) and 9–11 years (T3).

**Results:**

Three patterns derived at 6–8 years were broadly identified to be healthy, unhealthy and mixed patterns. Patterns at 9–11 years were dissimilar except for the unhealthy pattern. Individual behaviours characterising the patterns varied over time. No significant cross-sectional or prospective associations were observed with adiposity at both time points; however, children displaying the unhealthy pattern had higher adiposity measures than other patterns.

**Conclusion:**

Three non-identical patterns were identified at 6–8 and 9–11 years. The individual behaviours that characterised patterns (dominant behaviours) at both ages are possible drivers of the patterns obtained and could explain the lack of associations with adiposity. Identifying individual behaviour pattern drivers and strategic intervention are key to maintain and prevent the decline of healthy patterns.

Global prevalence of children with overweight and obesity continues to remain high. In Australia in 2017–18, at age 5, 25 % of children were with overweight and obesity (herein termed overweight)^(1)^. Childhood overweight has important public health implications as it tracks into adulthood and is associated with a range of negative health consequences such as increased cardiometabolic risk and poor mental health^(1)^.

Current evidence identifies a range of behavioural determinants, including children’s dietary, physical activity and sedentary behaviours, and sleep duration, that each play a significant role in overweight development in their growing years^(2–4)^. However, most previous evidence has examined these behaviours in isolation and has not considered the effects of these behaviours in combination. Owing to the complex multifaceted nature of overweight development, and the interaction of its behavioural determinants, novel approaches to understanding the genesis and prevention of child overweight would benefit from examining the integrated influence of these behaviours when compared with traditional single behaviour approaches^(5,6)^.

There exist many data-driven techniques to identify integrated patterns of these behaviours to subsequently test for associations with adiposity^(7,8)^. While varying patterns emerge, consisting of a number of behaviours co-occurring together, typically these can be classified as being healthy, unhealthy and mixed^(9)^. A recent systematic review in children aged between 5 and 12 years identified twenty-eight studies examining patterns comprising combinations of either diet, physical activity, sedentary behaviour and sleep^(9)^. Most frequently, healthy patterns were characterised by co-occurrence of high physical activity and low sedentary behaviour. The most prevalent unhealthy patterns were ‘high sedentary behaviour and low physical activity’, followed by ‘high sedentary behaviour and poor dietary behaviours’. Mixed patterns comprising high physical activity co-occurring with high sedentary behaviour were most prevalent^(9)^.

Three reviews^(3,9,10)^ have summarised the integrated influence of diet, physical activity, sedentary behaviour and/or sleep using behavioural patterns on adiposity in school-age children. Most studies investigated two or three behaviours (diet, physical activity and sedentary behaviour) using subjective measures, with physical activity and sedentary behaviour being most frequently investigated together. Studies that included sedentary behaviour predominantly focused on-screen use or television viewing, although other behaviours exist within this behaviour domain. Only six studies investigated patterns comprising all four behaviours and most included children aged over 9 years^(9)^. Five of these studies were cross-sectional^(5,11–14)^ and one longitudinal^(15)^. Evidence of cross-sectional and prospective associations with higher adiposity risk was most frequently reported for unhealthy patterns (low physical activity and high sedentary behaviour) compared to healthy (healthy diet and high physical activity)^(3,10)^ or mixed (low physical activity, low sedentary behaviour, poor fruit and vegetable consumption and high sleep duration) patterns^(9)^. Unhealthy patterns characterised by low physical activity and high sedentary behaviour; poor diet quality and low sleep duration; and snacking and sedentary behaviour showed stronger associations with later adiposity^(9)^. Evidence linking mixed patterns and adiposity risk remains inconclusive with studies reporting both high or low risk or no association^(9)^. Prospective studies and those investigating sleep in addition to diet, physical activity and sedentary behaviour to identify behavioural patterns are sparse in this age group, particularly in children between 5 and 9 years.

Patterns were previously derived cross-sectionally in children aged 6–8 years using three data reduction techniques (principal component analysis, cluster analysis, and latent profile analysis) to investigate and compare if the patterns derived were similar^(16)^. In this study, we aimed to identify patterns using a single technique (latent profile analysis), comprising diet, physical activity, sedentary behaviour and sleep data, in a longitudinal cohort of children aged 6–8 years, followed-up at 9–11 years, to investigate both cross-sectional and prospective associations of patterns with a single health outcome; adiposity. The inclusion of quiet play time and the use of both parent-reported and objectively measured data adds novelty to the present study.

## Methods

### Study design

**Fig. 1:**
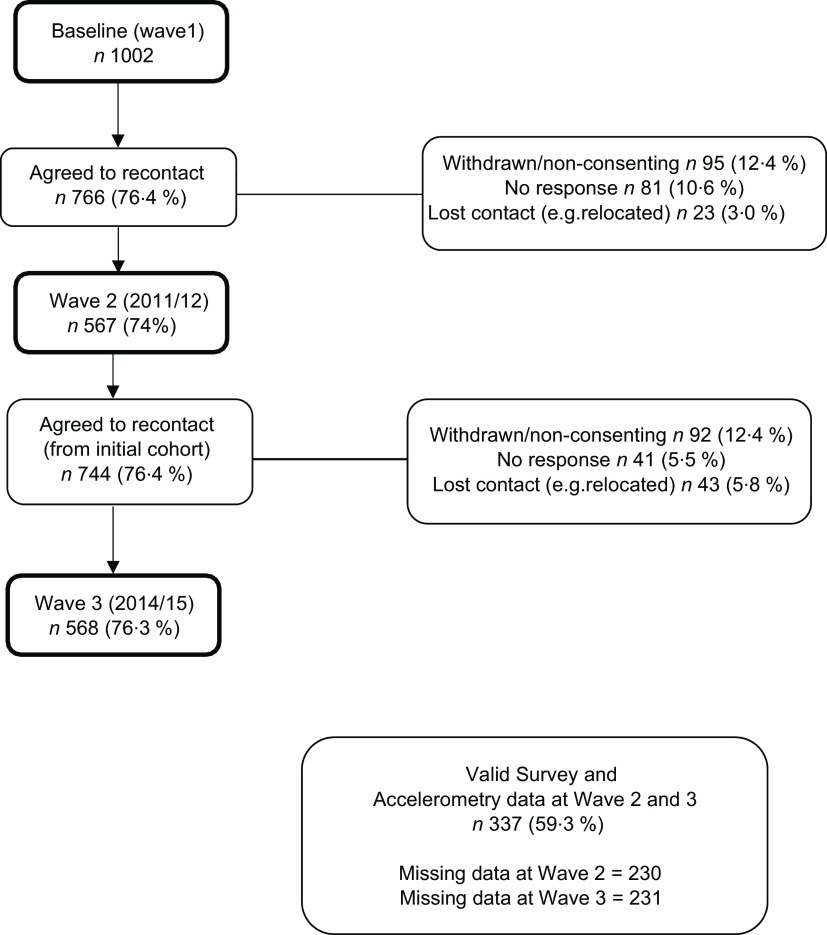
Participant flow diagram

Data were from the second (T2) and third (T3) waves of the Healthy Active Preschool and Primary Years (HAPPY) study, described in depth previously^(17)^. Briefly, the baseline sample consisted of 1002 parents of 3–5-year-old children in 2008–2009, recruited from preschools and early childcare centres. Parents were followed up twice, in 2011–2012 and 2014–2015 when children were aged 6–8 years (77 % retention rate) and 9–11 years (74 % retention rate), respectively. These details are summarised in Fig. 1. Information on dietary intake, physical activity, sedentary behaviour, sleep duration and adiposity outcomes was measured at both T2 and T3.

### Dietary intake

Dietary data were captured through parent-report using a validated 15-item FFQ^(18)^. A 7-point Likert scale (0–6 or more times) recorded the frequency of discretionary food item intakes in the previous week. There were six sweet and seven savoury discretionary food items, respectively. Sweet foods included spreads (peanut butter or Nutella), pre-sugared cereals, bakery items (sweet biscuits, cakes, muffins, doughnuts or fruit pies), lollies and snack bars, chocolate and ice-cream, while savoury foods included potato crisps or savoury biscuits, cheese and cheese spreads, pies and sausage rolls, pizza, hot chips or French fries, hot dogs and processed meats and takeaway foods. Frequency items were summed, then divided by seven to obtain daily sweet and savoury discretionary food intake values. Two items captured the frequency of daily fruit (fresh, canned, stewed or dried) and vegetable intake (raw or cooked) in the past 24 h, using a 6-point Likert scale (0–5 or more times).

### Physical activity, sedentary behaviour and sleep

Physical activity and sedentary behaviour were measured objectively using Actigraph GT1M uniaxial accelerometers (Pensacola, FL, USA). Accelerometers were hip-worn for eight consecutive days during waking hours and were removed for water-based activities. Accelerometer data recorded for a minimum of 8 hours a day for ≥ 4 d, including one weekend day, were considered valid. Counts > 2296/min^(19)^ and counts < 100/min^(20)^ were classified as moderate- to vigorous-intensity physical activity (MVPA) and sedentary time (ST), respectively. Residuals obtained by regressing accelerometer data on wear time were used to adjust MVPA and ST to total wear time.

Parents reported time (in hours and minutes) their children spent in specific physical activity and sedentary behaviours and sleep using a survey^(21)^. Organised sport duration was captured as time (total duration) spent in various organised sports (football, basketball, soccer, swimming, netball, gymnastics, dance, cricket and ‘other’ sports) for a typical week. Weekly total duration for each sport was summed to obtain the total weekly organised sport duration. Time spent playing outdoors was reported for a typical weekday and weekend day. Individual weekday (multiplied by five) and weekend day (multiplied by two) outdoor play duration values were summed to obtain the total weekly duration. The survey also captured the total number of hours during the week (Monday–Friday) and the weekend (Saturday and Sunday) that children engaged in the following sedentary behaviours: screen time (sum of television viewing and computer use excluding games), videogames (sum of computer games and handheld electronic games) and quiet playtime. Videogames were not included as part of screen time as children’s engagement in these behaviours can vary and have different effects on health^(16)^. Each sedentary behaviour was summed to obtain weekly durations. Total weekly duration for all activities divided by seven provided daily durations in minutes. Children’s usual nightly sleep duration was parent-reported in hours and minutes per night. Most survey items reported good reliability (ICC > 0·60)^(22)^, except for outdoor play (ICC = 0·44), screen time (ICC = 0·44) and quiet playtime (ICC = 0·10).

### Adiposity measures

Trained study staff followed standardised protocols to measure children’s height, weight and waist circumference either at school or at home. Weight was measured using a Wedderburn Tanita digital portable scale (to the nearest 0·1 kg), height using a Wedderburn Seca portable rigid stadiometer (to the nearest 0·1 cm) and waist circumference (to the nearest 0·1 cm) using a steel non-stretch tape. The tape was placed across the umbilicus, ensuring children were breathing naturally, and the stomach muscles were relaxed for the waist circumference measurement. Measurements were taken twice and a third time if there was a discrepancy (> 0·5 kg for weight, > 0·5 cm for height, > 0·1 cm for waist circumference) between the first two. The two closest values were averaged and used in the analyses^(23)^. BMI was converted to age and sex-specific BMI z-scores using IOTF cut-offs^(24)^. Children’s weight status categories (underweight or healthy, overweight and obese) were further derived using these cut points^(24,25)^.

### Covariates

Child age and sex and parent education (highest level of schooling) were reported by parents at both time points. Seven response options for parent’s highest level of schooling were categorised into university (university degree and post-graduate) and non-university education (no formal qualifications, year 10, year 12, trade/apprentice/certificate, diploma).

### Statistical analysis

Statistical analyses were conducted in Stata 16·0 (StataCorp, USA) and Mplus 8·0. Descriptive statistics summarised sample demographics and *t* tests/chi-square (for continuous/categorical variables, respectively) were used to test for differences between children included and excluded in the analyses. Twelve variables representing the behaviour domains: diet (four variables: sweet and savoury discretionary food intakes, fruit, vegetables), physical activity (three variables: MVPA levels, organised sport, and outdoor play duration), sedentary behaviour (four variables: screen, videogame, quiet play, and sedentary time) and sleep duration (1 variable) were standardised and included as inputs for a latent profile analysis to identify potential behavioural patterns, conducted separately for T2 and T3. The behavioural variables were converted to standard scores with a mean of 0 and sd of 1 for the latent profile analysis due to non-uniformity in the scales of the variables captured.

### Latent profile analysis

Latent profile analysis is a multivariate data reduction technique useful in summarising observations into latent profiles by assigning sample members with similar characteristics into mutually exclusive groups^(26)^. The technique utilises the underlying assumption that there are distinct unmeasured ‘latent’ profiles of (in this case) health behaviours in the sample population to which individuals belong. The number of groups to be derived must be specified by the investigator. The technique estimates the probability of sample members belonging to a particular group who are then typically assigned membership to the group with the highest probability^(26)^. A range of profile solutions (2–10 profiles) were derived and compared according to two recommended model fit estimation statistical criteria to determine the best-fitting model^(27)^. These were the Bayesian Information criteria (BIC; where lower values indicate better model fit)^(15)^ and the adjusted Lo-Mendel-Rubin (aLMR) test (assessing model fit improvement among adjoining profile models)^(26)^. Criteria values for the candidate models were compared to check for concordance in model fit using Mplus. The criteria suggested different profile solutions; nine (BIC) and three (aLMR) profile models at both time points (T2 and T3). Patterns from each model were compared for meaningfulness (how realistic), size and interpretability (having clear behavioural characteristics). The three-pattern model was more interpretable and was retained for both time points. Estimated mean values of behaviour standardised scores for each pattern were examined and a cut point of ±0·2 was used^(16)^ to identify behaviours that were comparatively high or low in the patterns derived. Due to the data-driven nature of pattern derivation techniques, the patterns identified in this analysis using latent profile analysis were non-identical to those derived in our previous analysis^(16)^.

Multiple linear regression tested cross-sectional associations between obtained behavioural patterns (categorical independent variable) and adiposity measures (BMI z-score and waist circumference; continuous dependent variables) at T2 and T3, respectively. Additional multiple linear regression models examined associations between behavioural patterns at T2 and changes in adiposity measures from T2 to T3 (adjusted for baseline adiposity). Overall effects of the patterns on adiposity measures were assessed using Wald tests, and where there was evidence of differences between groups (at the *P* < 0·05 level) then pairwise comparisons were examined. All models were adjusted for parent education and included cluster-robust standard errors (using the ‘sandwich’ estimator)^(28)^ to account for potential clustering by recruitment centre. Relevant linear regression assumptions were assessed (e.g. normality was explored using histograms and Q–Q plots of the residuals, multi-collinearity using variance inflation factors, and homoscedasticity using residuals *v*. fitted values plots and the information matrix test) and found to be met. Waist circumference models were additionally adjusted for child age and sex, as unlike BMI z-scores these were not age and sex normed.

## Results

Complete data were available for 337 children at both time points. Children were excluded if they had missing behavioural and adiposity data at both time points. No differences in child age, sex and BMI were observed between excluded and included children; however, those excluded (53·5 %) had a larger proportion of tertiary-level educated parents than those included (46·5 %) at both time points. Sample characteristics are described in Table 1. The mean age of children at T2 and T3 were 7·5 years and 10·5 years, respectively. Most children (> 80 %) were classified within the healthy weight and underweight category at T2 and T3. Differences in parent education, waist circumference, weight status, fruit and vegetable intake, organised sport duration, MVPA, screen, sedentary time and sleep duration were observed between T2 and T3.

**Table 1:** Characteristics of the HAPPY sample at T2 and T3 (*n* 337)

Variable	T2			T3		
	*n*	%	Range	*n*	%	Range
Child age (years)						
Mean	7·5		6·0–9·1	10·6		9·0–12·2
sd	0·7			0·7		
Sex						
Male	188	55·8	–	–		–
Female	149	44·2				
Parent education*						
Below university level	109	32·3	–	107	31·8	–
University and above	228	67·7		230	68·2	
BMI z-score						
Mean	0·40		−2·5–2·9	0·33		−3·2–3·3
sd	0·9			1·1		
Waist circumference (cm)*						
Mean	58·3		27·9–85·0	65·5		49·2–112·5
sd	5·6			8·4		
Weight status*			–			–
Underweight	12	3·6		19	5·6	
Healthy	280	83·1		255	75·7	
Overweight	42	12·4		55	16·3	
Obese	3	0·9		8	2·4	
	Mean	sd		Mean	sd	
Diet						
Fruit intake (times/d)*	2·3	1·3	0·0–5·0	2·6	1·3	0·0–5·0
Vegetable intake (times/d)*	2·9	1·3	0·0–5·0	3·4	1·2	0·0–5·0
Sweet discretionary food intake (times/d)	1·5	0·7	0·0–3·7	1·5	0·7	0·0–3·9
Savoury discretionary food intake (times/d)	1·2	0·5	0·0–2·6	1·1	0·5	0·0–3·0
Physical activity						
Organised sport (min/d)*	23·3	19·9	0·0–197·1	35·9	29·1	0·0–142·1
Outdoor play (min/d)	142·3	75·7	6·4–454·3	148·0	82·7	0·0–548·6
MVPA (min/d)*,†	108·7	29·4	43·7–206·2	61·8	24·8	9·6–139·8
Sedentary behaviour						
Screen time (min/d)*	93·4	57·3	0·0–321·4	152·1	91·0	0·0–600·0
Videogames (min/d)	23·5	32·2	0·0–214·3	22·9	50·6	0·0–445·7
Quiet play time (min/d)	53·6	41·2	0·0–342·9	53·7	36·1	0·0–214·3
Sedentary time (min/d)*,†	359·7	49·1	234·5–512·6	440·2	50·4	288·6–603·2
Sleep duration (min/d)*	625·9	46·8	480·0–720·0	588·8	52·2	390·0–740·0

MVPA; moderate- to vigorous-intensity physical activity, T2; wave two; T3, wave three.

* Indicates significant differences between values/proportions at T2 and T3.

† Mean accelerometer wear time was 702 and 744 min per day for T2 and T3 time, respectively.

Characteristics of behavioural patterns identified are presented in Table 2. At T2, the three patterns identified were labelled as (1) *unhealthy* (*n* 72), characterised by lowest consumption of fruit and vegetables, low overall physical activity and sleep duration, and highest sweet discretionary food intake and overall sedentary time; (2) *non-sedentary healthy eaters* (‘healthy profile’, *n* 195), comprised highest consumption of fruit and vegetables, lowest consumption of sweet discretionary food items and lowest screen time and (3) *active unhealthy eaters* (‘mixed profile’, *n* 70), characterised by high discretionary food intake, high outdoor play duration and MVPA, low fruit consumption and lowest sedentary time.

**Table 2:** Pattern characteristics* for LPA at T2 (6–8 years) and T3 (9–11 years)

	Variables	T2			T3		
		Unhealthy (*n* 72)	Non-sedentary healthy eaters (*n* 195)	Active unhealthy eaters (*n* 70)	Unhealthy (*n* 52)	Intermediate (*n* 222)	Active and non-sedentary (*n* 63)
Diet	Fruit intake	−0·63	0·32	−0·20	−0·63	0·12	0·08
	Vegetable intake	−0·65	0·25	−0·01	−0·48	0·10	0·09
	Sweet discretionary food intake	0·38	−0·23	0·23	−0·01	−0·02	0·11
	Savoury discretionary food intake	0·04	−0·19	0·47	0·05	−0·04	0·11
PA	Organised sport	−0·29	0·15	−0·10	−0·70	0·01	0·57
	Outdoor play	−0·42	0·06	0·24	−0·61	0·01	0·42
	MVPA	−1·16	−0·08	1·38	−1·24	−0·14	1·52
SB	Screen time	0·52	−0·23	0·09	0·66	−0·15	0·00
	Videogame time	0·26	−0·04	−0·15	0·79	−0·18	−0·01
	Quiet play time	0·25	−0·06	−0·10	−0·22	0·08	−0·10
	Sedentary time	1·06	0·08	−1·29	1·33	0·06	−1·31
Sleep	Sleep duration	−0·42	0·13	0·04	−0·73	0·14	0·10

LPA; latent profile analysis, T2; wave two, T3; wave three, MVPA; moderate- to vigorous-intensity physical activity.

* Values presented are estimated mean standard scores of behavioural variables for each latent profile (values > or < ±0·2 were used to identify behaviours that are high or low, respectively).

At T3, three patterns were identified, and with the exception of the unhealthy pattern, these differed from the patterns identified at T2. These patterns were labelled as (1) *unhealthy* (*n* 52), characterised by lowest fruit and vegetable intake, lowest overall physical activity and sleep duration with highest overall sedentary behaviour; (2) *intermediate* (‘slightly healthy profile’, *n* 222), comprised children exhibiting behaviours that were not distinctively high/low as most behaviours in this pattern were close to the sample average and (3) *active and non-sedentary* (‘healthy profile’, *n* 63), comprising highest organised sport duration, outdoor play and MVPA and lowest sedentary time.

Although pattern types identified (healthy/unhealthy/mixed) were somewhat consistent across T2 and T3, there was little consistency in the individual behaviours that feature in the healthy and mixed patterns, whereas those in the unhealthy patterns were relatively consistent. Furthermore, the number of children that remained in similar pattern types from T2 and T3 was inconsistent. About half of the children from T2 remained in the unhealthy profile at T3 (Table 3). Greatest movement of participants was observed between the other two sets of patterns, with the healthy pattern being most dominant at T2 and the intermediate pattern at T3. A chi-square test for independence indicated that T2 and T3 pattern memberships were related (*P* < 0·0005), with ‘unhealthy’ individuals at T2 also being in the ‘unhealthy’ pattern at T3 at a rate greater than chance (standardised adjusted residual = 7·3), ‘non-sedentary healthy eaters’ at T2 being in the ‘intermediate’ group at T3 at a rate greater than chance (std. adj. residual = 4·3) and ‘active unhealthy eaters’ at T2 being in the ‘active and non-sedentary’ pattern at T3 at a rate greater than chance (std. adj. residual = 6·9).

**Table 3:** Distribution and shifts of the number of children between behavioural patterns at T2 and T3

	Patterns at T3			
Patterns at T2	Unhealthy	Intermediate	Active and non-sedentary	Total
Unhealthy	31	40	1	72
Non-sedentary healthy eaters	19	147	29	195
Active unhealthy eaters	2	35	33	70
Total	52	222	63	337

Mean values of adiposity measures at T2 and T3 and the change in these measures from T2 to T3 are presented in Table 4. At T2 and T3, the mean BMI z-scores and waist circumferences were similar across the patterns, except for those in the unhealthy pattern at T3. The highest BMI z-score and the largest waist circumference were observed for those in the unhealthy pattern at both T2 and T3. The children classified in the unhealthy pattern at T2 displayed the biggest reduction in BMI z-scores from T2 to T3. At all-time points, Wald’s test did not provide evidence of differences between patterns (*P* > 0·05) in BMI z-score or waist circumference.

**Table 4:** Associations of behavioural patterns and adiposity at T2 (6–8 years) and T3 (9–11 years)

Adiposity measures	Behaviour patterns – T2						
	Unhealthy (*n* 72)		Non-sedentary healthy eaters (*n* 195)		Active unhealthy eaters (*n* 70)		
	Mean	sd	Mean	sd	Mean	sd	Group difference*
T2 BMI z-score	0·39	1·0	0·39	0·9	0·42	0·9	F (2, 111) = 0·02, *P* = 0·98
T2 waist circumference (cm)	59·0	6·8	58·2	5·5	57·8	4·6	F (2, 111) = 0·10, *P* = 0·91
T2–T3 change in BMI z-score	−0·11	0·5	−0·06	0·5	−0·01	0·5	F (2, 111) = 0·73, *P* = 0·48
T2–T3 change in waist circumference (cm)	6·5	5·0	7·5	5·8	7·2	4·2	F (2, 111) = 1·44, *P* = 0·24

Adiposity measures	Behaviour patterns – T3						
	Unhealthy (*n* 52)		Intermediate (*n* 222)		Active and non-sedentary (*n* 63)		
	Mean	sd	Mean	sd	Mean	sd	Group difference*
T3 BMI z-score	0·46	1·2	0·31	1·1	0·31	1·0	F (2111) = 0·28, *P* = 0·76
T3 waist circumference (cm)	66·9	10·8	65·5	8·4	64·4	6·1	F (2111) = 2·12, *P* = 0·13

T2; wave two, T3; wave three.

* Test of overall group effects for linear regression models adjusted for maternal education and clustering by recruitment centre. Models for waist circumference outcomes were additionally adjusted for child age and sex.

## Discussion

This paper identified behavioural patterns in children aged 6–8 years and 9–11 years to subsequently investigate cross-sectional and prospective associations of these patterns with adiposity. Using latent profile analysis, three behavioural patterns were identified at both time points. At T2, the patterns identified were broadly classified as healthy, unhealthy and mixed; however, these patterns were not identical at follow-up, suggesting a change in behavioural patterns as children age. Little evidence of associations was observed between patterns and adiposity cross-sectionally or prospectively.

Consistent with our findings at T2, three studies using cluster analysis have reported a similar healthy behavioural pattern comprising a healthy diet and low screen time^(5,29)^ or low sweetened beverage intake and low sedentary behaviour^(30)^ in children. The co-occurrence of healthy dietary behaviours and low screen time is an important finding as it is less prevalent in prior studies, and most frequently, these behaviours co-occur as unhealthy patterns comprising high screen time and high discretionary food intake^(3,9,10)^. At T3, a different healthy pattern comprising high physical activity and low sedentary behaviour, although exhibited by a smaller group of children, is consistent with being the most prevalent healthy pattern in the literature for this age group^(9)^.

Consistency of patterns over time has been reported by two previous studies (across two time points) in children aged 5–12 years, one using cluster analysis^(31)^ and the other using latent class analysis^(32)^. An additional study, however, in younger children (≤ 5 years) found two similar patterns at three time points using principal component analysis^(33)^. The relative stability of behaviours in younger children and the use of different methods might explain the inconsistency in findings. Comparing the present study’s healthy patterns at T2 and T3, it appears that different individual behaviours may drive patterns at different ages. Although tracking was not assessed, the differences in the prominence of dietary and physical activity behaviours within the healthy patterns at different time points suggest that younger children eat healthier diets whereas older children engage in more physical activity. This seems plausible given children’s growing independence in making food choices may result in less healthy diets, and they become more competent in engaging in physical activity with age^(34)^. The largest intermediate behavioural pattern at T3 included 40 % of children who displayed the healthy pattern at T2, possibly highlighting a combination of the change in behavioural grouping along with a decline in healthy behaviours by age. Further prospective studies are warranted to confirm consistency in behavioural pattern changes with age, across different studies using latent profile analyses.

In contrast to the healthy patterns, the unhealthy patterns were quite similar at both time points with children displaying health-demoting behaviours across all four behaviour domains. Similar patterns characterised by low physical activity, low sleep duration, poor diet and high screen time were reported by two studies in children aged 9–12 years^(11,14)^. Unhealthy patterns appear most robust, evidenced by the consistency of unhealthy patterns with previous literature, and the stability of the unhealthy pattern in both age groups in this study sample. Therefore, further investigation of the children exhibiting these unhealthy patterns is warranted to tailor intervention efforts. The current study extends findings and adds novelty from previous studies by confirming these patterns with a different analytic approach, using comprehensive data for each behaviour, as well as the addition of objectively assessed physical activity data.

The mixed pattern identified at T2 has not been reported previously. The co-occurrence of high physical activity, high discretionary food intake along with low screen time in the mixed pattern suggest possible mechanisms of compensating energy expenditure due to high physical activity^(35)^ through the intake of energy-dense discretionary food. Most prevalent mixed patterns in cross-sectional studies comprise behaviours being synchronously high or low across behaviours investigated^(9)^, whilst the pattern in our study displayed asynchronous (high and low) behaviours. Prospectively, consistent mixed patterns were identified when children were 6 and 9 years in a study using a latent transition model; however, their patterns comprised only two behaviours (physical activity and sedentary behaviour)^(32)^. Furthermore, two other studies that identified similar patterns over time^(31,33)^ also investigated fewer behaviours compared to those included in the present study. This suggests that the number and variation in behavioural variables included might influence if consistent patterns are observed over time, with fewer behaviours included showing better consistency. Our study included twelve behavioural variables in the pattern analyses including some specific behaviours not frequently explored such as quiet play and video games which might add to the lack of consistent patterns seen across time. Despite these methods being data-driven, making the patterns derived being sample specific, the analytical method used and the number of behaviours included influence the differences in patterns across studies and highlight that individual behaviours are possible drivers of the differences in patterns across time.

Although similar number of patterns were identified at T2 and T3, distinct patterns characterised by different behaviours were revealed. Variation in intakes of fruit and vegetables and discretionary items were more prominent in younger than older children, especially for consumption of discretionary food items, suggesting that older children eat a less healthy diet over time. For sedentary behaviour, time spent in videogames was more varied for older children, and conversely, lesser variation was observed for quiet play time. Higher time spent playing videogames in older children is consistent with the increase in screen media use as children age, with less time spent in more passive sedentary behaviours (e.g. quiet play)^(36)^. Physical activity was discriminant at both age periods, but the scores from the latent profile analysis indicated that those in the unhealthy patterns for both younger and older children displayed declines in physical activity, suggesting worsening of physical activity levels by age^(37)^. These discriminant individual behaviours are important as they drive the differences seen in patterns derived across time as identified in our cohort and may help understand subsequent associations seen with health outcomes.

Given that associations between individual behaviours and adiposity are established^(11)^, associations of pooled behavioural patterns and adiposity were expected. However, cross-sectionally and prospectively, there was little evidence of associations between behavioural patterns and adiposity. The relatively low number of children consistently displaying a particular pattern type across time might contribute to the lack of power to detect associations. Studies examining associations between patterns (comprising all four behaviour domains) and adiposity are few and the evidence is unclear^(9)^. Two cross-sectional studies each identified a similar unhealthy pattern but showed discrepant findings to the current study. One study^(11)^ found a lower prevalence of children with overweight in their unhealthy pattern compared to the mixed patterns in their study and the other^(14)^ did not find any differences. Other studies reported lower adiposity risk for their healthy patterns^(5,11,12)^, and high^(11)^ and low^(11,13)^ prevalence of children with overweight in their mixed patterns, compared to other patterns they identified. Prospectively, only one study showed greater adiposity for a high sedentary behaviour and low physical activity pattern, and a lower sleep duration and high discretionary food intake pattern, compared to their healthy pattern^(15)^. Patterns comprising two behaviours show stronger evidence of associations with adiposity than those with three or more behaviours, with stronger evidence seen for unhealthy patterns over healthy and mixed patterns^(9)^. This accentuates the complexity in understanding the synergistic contribution of these individual behaviours towards overweight genesis when three or more behaviours are at play. Additionally, the variation in the individual behaviours that comprise patterns and the mixed associations with adiposity across studies might suggest that behaviours that characterise/dominate patterns might be drivers of the differences in the evidence of associations and may explain our study findings.

A major strength of this study is the inclusion of sleep time in deriving patterns, given previous studies have focused primarily on diet, physical activity and sedentary behaviour. The inclusion of quiet playtime and videogame use is also novel as these behaviours are less frequently investigated within patterns, and previous evidence focuses on assessing television viewing or screen time as a proxy for sedentary behaviour. An additional strength is the inclusion of both objective (accelerometry) and self-reported behavioural data, and the longitudinal design of the analyses as most studies assessing all four behaviour domains were cross-sectional. The use of latent profile analysis is an additional strength, as it provides individual probability of belonging to a particular pattern and the likelihood of an individual displaying those pattern characteristics^(26,38)^. The objective measurement of adiposity measures is a further strength as most previous literature have used self-reported measures^(9)^.

Limitations include most behavioural variables being captured through subjective measures. Nonetheless, these measures are well established, reliable and practical in large community-based studies where objective assessment of dietary intakes are not feasible. Some measures had relatively low test–retest reliability (quiet, outdoor play and screen time), but were still included due to expected week to week variation. These variables provide information on relevant context-specific behaviours which cannot easily be measured objectively. Further prospective studies examining all four behaviour domains and associations with adiposity, using objective methods where possible, are warranted. Latent profile analysis being a data-driven method makes the patterns identified sample-specific and not generalisable to the wider Australian population. Nonetheless, this cohort included a large sample of children recruited from low-, mid- and high-socio-economic areas and had parent education levels (68 % at T2 and 70 % at T3) that were comparable to the population level (58 %) for post-secondary qualifications^(39)^. It’s worth noting the limitation of using BMI z-scores to track changes in adiposity over time as a previous study^(40)^ found BMI z-score to be a poor predictor of body fat in children with obesity. Lastly, the lower proportion of overweight children in the study sample compared to the national average (25 %) limits the generalisability of the study findings to this population and subsequent conclusions about obesity in this age group. This might also explain the lack of associations between behavioural patterns and adiposity in this cohort.

### Conclusion

Three non-identical behavioural patterns involving four behaviour domains were identified in children aged 6–8 and 9–11 years. Patterns were more distinct in younger children than older children. Individual behaviours that were discriminant across time points appeared to drive the differences in patterns by age. Across patterns and over time, the observed decline in healthy behaviours and increase in unhealthy behaviours indicates the need to monitor all behavioural patterns by age without solely focussing on those children displaying unhealthy patterns. Despite little evidence of differences in adiposity measures between patterns, children in the unhealthy patterns displayed higher BMI z-scores and waist circumferences than those in the other patterns at both time points and may require monitoring for worsening of adiposity with age. Changes in behavioural patterns and the individual behaviours that drive them are important to identify key behaviours and time points to strategically intervene to help children maintain healthy patterns or improve unhealthy patterns as they age.
